# Intrastent Restenosis: A Comprehensive Review

**DOI:** 10.3390/ijms25031715

**Published:** 2024-01-30

**Authors:** Ioan-Teodor Bajeu, Adelina-Gabriela Niculescu, Alexandru Scafa-Udriște, Ecaterina Andronescu

**Affiliations:** 1 Department of Science and Engineering of Oxide Materials and Nanomaterials, Faculty of Chemical Engineering and Biotechnologies, National University of Science and Technology Politehnica Bucharest, 1-7 Polizu St., 011061 Bucharest, Romania; tdr.bajeu@gmail.com (I.-T.B.); adelina.niculescu@upb.ro (A.-G.N.); ecaterina.andronescu@upb.ro (E.A.); 2 Department of Cardiology, Clinical Emergency Hospital of Bucharest, Calea Floreasca 8, 014461 Bucharest, Romania; 3 Research Institute of the University of Bucharest—ICUB, University of Bucharest, 90 Panduri Road, 050657 Bucharest, Romania; 4 Department Cardio-Thoracic Pathology, University of Medicine and Pharmacy Carol Davila, Eroii Sanitari Bvd. 8, 050474 Bucharest, Romania; 5 Academy of Romanian Scientists, Ilfov No. 3, 050044 Bucharest, Romania

**Keywords:** intrastent restenosis, bare-metal stents, drug-eluting stents, pathophysiology, intravascular imaging, risk factors, coronary artery disease, treatment, drug-eluting ballons, angiography

## Abstract

The primary objective of this paper is to delineate and elucidate the contemporary advancements, developments, and prevailing trajectories concerning intrastent restenosis (ISR). We aim to provide a thorough overview of the most recent developments in this area, covering various aspects such as pathophysiological insights, therapeutic approaches, and new strategies for tackling the complex challenges of ISR in modern clinical settings. The authors have undertaken a study to address a relatively new medical challenge, recognizing its significant impact on the morbidity and mortality of individuals with cardiovascular diseases. This effort is driven by the need to fully understand, analyze, and possibly improve the outcomes of this emerging medical issue within the cardiovascular disease field. We acknowledge its considerable clinical implications and the necessity for innovative methods to mitigate its effects on patient outcomes. Therefore, our emphasis was directed towards elucidating the principal facets of the condition’s prevalence, expounding upon the foundational mechanisms underscoring conspicuous restenosis, and delineating the risk factors relevant in shaping the contemporary landscape of diagnostic and therapeutic modalities. This thorough examination aims to provide a comprehensive understanding of the various dimensions of the condition, including epidemiological data, pathophysiological complexities, and clinical considerations critical for evaluating and enhancing current diagnostic and treatment approaches.

## 1. Introduction

Restenosis refers to the narrowing of a blood vessel’s diameter following an angioplasty procedure [1]. Intrastent restenosis (ISR) is a challenging medical problem [2]. A meta-analysis showed that percutaneous coronary intervention (PCI) for ISR is associated with a higher incidence of adverse cardiac events than PCI for de novo lesions. This happens especially because of a higher incidence of risk-adjusted major adverse cardiac events compared with PCI for de novo lesions at a median of ≈30 months [3]. 

Although it is a lesser encountered problem than before due to the use of drug-eluting stents, it continues to play a major role in modern medical practice because of extensive stents being implanted in current practice. The 2020 National Cardiovascular Data Registry, including 5,100,394 patients, shows that reinterventions for ISR were necessary for 542,112 patients, representing 10.6 percent of all the PCIs [4].

It is essential to know that ISR incidence depends on a series of variables such as the stent type, patient population, cardiovascular risk factors, medication used, and follow-up duration [5]. When it comes to the stents used, it was reported that the incidence of ISR ranged from 5 to 30 percent in patients with bare-metal stents (BMSs) [6]. Introducing drug-eluting stents (DESs) has significantly reduced the ISR rates to 2 to 10 percent [7]. Despite these results, due to millions of DESs used for treating cardiovascular problems, it can be said that ISR is a public health problem worldwide [8].

In this context, this review aims to underscore the primary challenges engendered by ISR, recognizing its consequential influence on individuals with a prior history of coronary angioplasty. The paramount objective is to traverse the pathophysiological intricacies inherent to restenosis, thereby seeking a comprehensive understanding of the underlying issues. In doing so, we aim to navigate through diverse diagnostic modalities and therapeutic interventions, propounding a nuanced exploration of approaches to mitigate the adverse impact of restenosis in individuals who have undergone coronary angioplasty procedures.

## 2. ISR—Definition, Incidence, and Pathophysiology

ISR is characterized by a progressive luminal constriction within the stent, predominantly manifesting within the timeframe of 3 to 12 months subsequent to stent angioplasty [9]. From a clinical perspective, this phenomenon manifests as recurrent angina or heart failure [10]. It is noteworthy that it may also manifest as acute myocardial infarction in approximately 10% of afflicted patients [7]. In contrast, intrastent thrombosis constitutes an acute thrombotic occlusion, representing a dire event with the potential for precipitating sudden cardiac death or protracted acute myocardial infarction [11]. Despite the early revascularization, the mortality at 6 months is very high in this case [9].

The incidence of ISR is contingent upon various factors, including the stent type, the intricacy of the stented lesions, and the presence of risk factors [5]. Significantly elevated restenosis rates are observed in instances characterized by lesion complexity, such as those involving small vessels, extended lesions, or bifurcation lesions [12]. Determining the incidence of ISR is challenging due to its dependence on a multifactorial and variable interplay of factors. During the period in which balloon angioplasties were used, restenosis rates ranged from 32% to 55% among all procedures, subsequently declining to a range of 17% to 41% [13,14,15,16] with the advent of bare-metal stents [17,18,19]. A pivotal achievement in addressing this complication was the adoption of DESs, leading to a reduction in restenosis rates to levels below 10% [20,21]. ISR seems to manifest more frequently in patients suffering from multivessel disease compared to those affected solely by single-vessel disease [22].

ISR refers to a condition characterized by a significant reduction, typically equal to or exceeding 50%, in the diameter of the coronary lumen [23].

The advent of coronary stents has substantively transformed the therapeutic landscape for individuals afflicted by both chronic coronary syndromes and acute coronary syndromes [24,25]. Nevertheless, the widespread adoption of percutaneous revascularization procedures has ushered in a novel pathological phenomenon: ISR. The pathophysiology of ISR is a complex process involving many cellular mechanisms and depends on a series of risk factors [26].

It is characterized by the re-narrowing of a coronary artery at the site of a previously implanted stent [27]. The predominant mechanism underlying ISR is characterized by tissue proliferation, commonly referred to as neointimal hyperplasia [28]. This process reflects an exaggerated homeostatic healing response triggered by the arterial wall injury incurred during stent implantation, as the literature delineates [29]. The causative factors implicated in this phenomenon encompass localized inflammation arising from mechanical injury to the intimal and medial layers. This subsequently drives an aggressive neointimal hyperplasia marked by the proliferation of smooth muscle cells and the deposition of extracellular matrix [30,31]. Moreover, hypersensitivity reactions to the metallic components and polymer constituents intrinsic to early-generation DESs have been established as recognized mechanisms contributing to neointimal hyperplasia [32].

Coronary atherosclerotic disease is most commonly managed through the intervention of angioplasty with stent placement, a therapeutic modality that, while lifesaving, has been associated with adverse events that curtail its enduring efficacy [33]. Technical inadequacies, exemplified by factors such as stent malposition or inadequate expansion, resulting in the establishment of laminar blood flow patterns, subsequently promote the development of neointimal hyperplasia [34]. After angioplasty with a stent, a prevailing concern in long-term progression is the occurrence of intrastent neo-atherosclerosis [35]. Notably, the utilization of old-generation bare-metal stents has been constrained in adherence to contemporary medical practice guidelines due to a notable incidence of ISR, which has historically ranged from 17% to 41% [10]. While the introduction of new-generation DESs has substantially reduced the rate of such complications [20], DES-related failures persist as a matter of concern, affecting up to 10% of all implanted stents [20,21]. 

**Figure 1:**
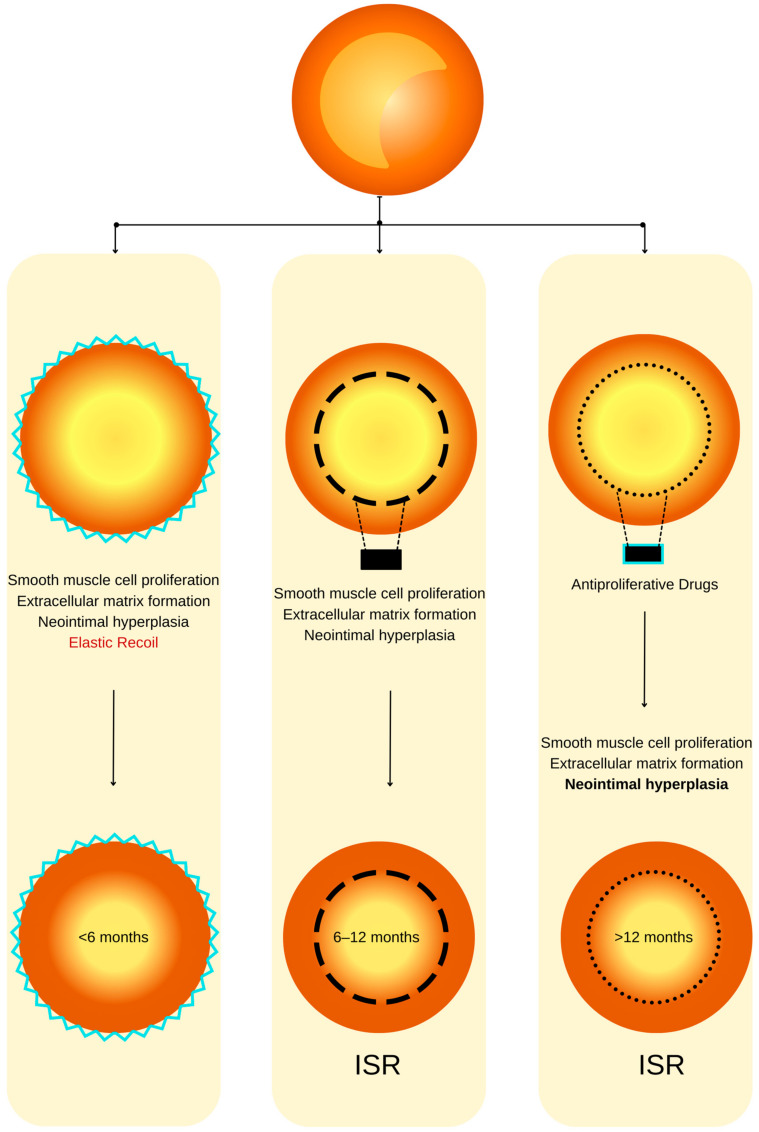
ISR physiopathology (adapted from an open–access source [32]). (**Left**): Coronary restenosis after conventional balloon angioplasty primarily arises from the phenomenon of elastic recoil of the arterial vessel wall. Moreover, the trauma inflicted on the coronary artery triggers the initiation of smooth muscle cell proliferation, their migration, and the deposition of an extracellular matrix. This cascade of events culminates in the formation of neointimal hyperplasia, which ultimately contributes to the pathogenesis of restenosis. (**Center**): The deployment of a BMS is associated with a heightened level of vascular injury, thereby augmenting the magnitude of neointimal hyperplasia and elevating the risk of in-stent restenosis. (**Right**): DESs dispense antiproliferative agents, effectively mitigating the extent of neointimal hyperplasia and correspondingly diminishing the susceptibility to in-stent restenosis.

**Figure 2:**
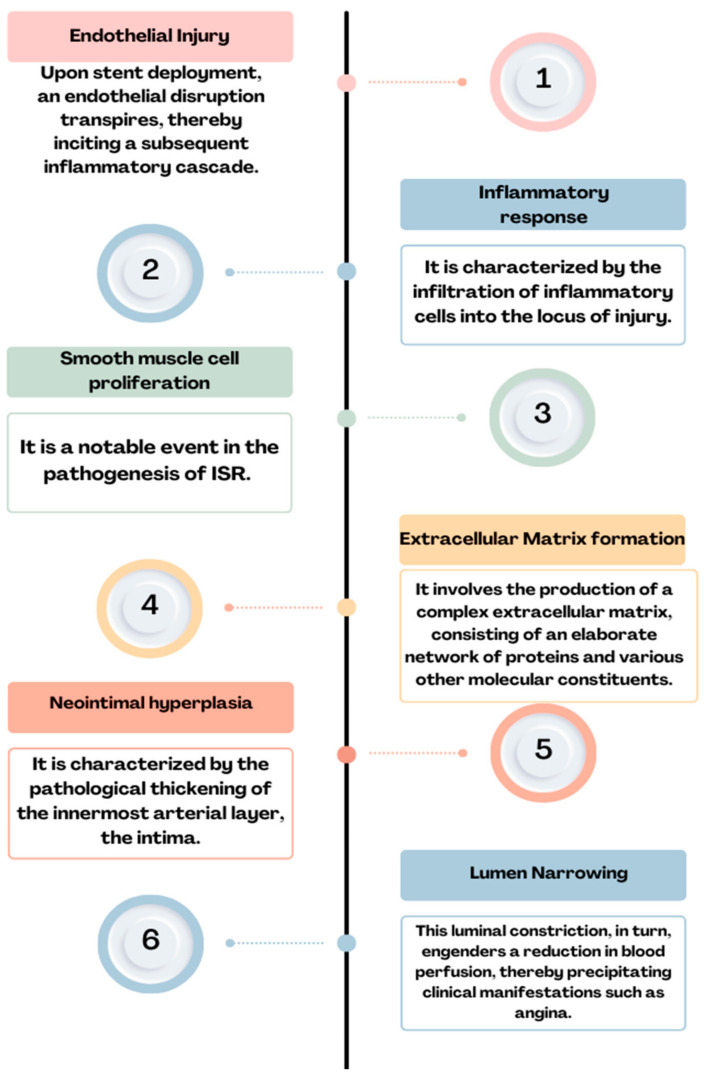
Physiopathology of ISR: endothelial injury→ inflammatory response→ smooth muscle cell proliferation→ extracellular matrix formation→ neointimal hyperplasia→ lumen narrowing = ISR.

As we mentioned before, using BMSs leads to a high rate of restenosis that can reach 40%. The principal etiology of this complication is commonly attributed to the incremental expansion of the extracellular fibrotic matrix, resulting in a gradual reduction in the luminal diameter [20]. In the contemporary era marked by the utilization of DESs, the pathogenesis of ISR appears to be contingent upon several interrelated factors, including the host’s heightened material sensitivity, the suppression of healing processes due to antiproliferative medications, and the organism’s reaction to stent implantation [36,37] (Figure 1). The pathophysiology of ISR (Figure 2) appears to involve a complex cascade of events, including the hyperplasia of smooth muscle cells, the migration of pro-inflammatory cells, the recruitment of marrow progenitors, including bone-marrow-derived progenitor cells (BMPCs), and the proliferation of the extracellular matrix, as suggested by the existing literature [36,37]. Hence, the interplay between the systemic pro-inflammatory response and the concurrent local pro-inflammatory response induced by the endothelial disruption during stent implantation collectively orchestrates the vascular healing process [38]. This dynamic interaction culminates in forming a neointimal zone characterized by the proliferation of unregulated smooth muscle fibers, ultimately contributing to the clinical manifestation of restenosis [39]. At the local level, the injury induced by stent deployment initiates a multifaceted cascade of processes, commencing with endothelial disruption and subsequent exposure of the intimal layer, which imparts a prothrombotic effect [38]. This local inflammatory milieu subsequently triggers the release of cytokines and growth factors, activates platelets, and fosters the proliferation and migration of smooth muscle fibers. These alterations can culminate in two potential outcomes: vascular healing or pathological progression. The latter pathological process, in particular, may eventually predispose individuals to ISR. Endothelial activation, prompted by cellular injury induced by the mechanical impact of the stent, initiates a cascade of events, including platelet activation. Notably, platelets are often the primary cells to respond to stent placement [40,41].

After arterial injury, platelets promptly adhere to the affected site and initiate the release of thromboxane A2. The glycoprotein (GP) IIb/IIIa complex engages with fibrinogen, facilitating platelet aggregation and activation [42]. Furthermore, activated platelets release many bioactive factors, among which the platelet-derived growth factor (PDGF) is prominent. PDGF exhibits both mitogenic and chemotactic properties, significantly influencing smooth muscle cells [43]. It contributes to the induction of oxidative stress, thereby facilitating the transition of smooth muscle cells from a contractile phenotype to a synthetic one [44]. The release of histamine from mast cells and platelets has been implicated in the pathogenesis of intimal hyperplasia [44]. This assertion supports studies involving porcine models of endothelial dysfunction, wherein a substantial 20- to 90-fold elevation in histamine concentration has been documented [45]. Interleukin-1, when released by platelets, triggers an upregulation in the production of interleukin-6 and interleukin-8, exerting pro-inflammatory effects. This cascade of events includes the stimulation of smooth muscle fiber migration, achieved through the activation of actin polymerization and the initiation of tyrosine phosphorylation at the cytoskeletal protein level, particularly in association with focal adhesion and smooth muscle fiber proliferation [38]. Extracellular vesicles emanating from platelets elicit a response in smooth muscle fibers, inducing the production of interleukin-6 while concurrently triggering the expression of αIIbβ3 and P-selectin. These cellular changes promote interactions between smooth muscle fibers and monocytes [46].

Furthermore, platelet-derived microvesicles have been demonstrated to induce endothelial protein C receptor (EPCR) proliferation characterized by the presence of von Willebrand factor (vWF+) and CD34+ markers. This effect is mediated through the release of transforming growth factor (TGF)-β1, as evidenced in a rat arterial injury model [47].

Within the vessel wall, the coexistence of an inflammatory response stemming from the presence of the foreign body, coupled with the anti-inflammatory effect exerted by the drug released from the stent, collectively engenders a deceleration in the process of reendothelialization. The exposure of adhesion molecules, such as P-selectin, serves as a stimulus for the recruitment of corresponding monocytes and the subsequent secretion of pro-inflammatory cytokines, namely interleukins 6 and 8. This cascade of events culminates in the infiltration of neutrophils, monocytes, and macrophages into the subendothelial space [38]. Elevated levels of monocytes and eosinophils at the three-month mark following PCI serve as predictive factors for late ISR after deploying a pharmacologically active stent [48]. While the precise involvement of mast cells in the pathophysiology of restenosis remains a subject of ongoing investigation, it is noteworthy that mast cells have been observed to release chymase [49], an enzyme implicated in generating angiotensin II and tumor growth factor-β. This consequential cascade of molecular events subsequently fosters fibroblast proliferation and is associated with the development of neointimal formation [50,51]. Experimental evidence in animal models has demonstrated the efficacy of chymase expression inhibitors in attenuating neointimal formation [52].

Neo-atherosclerosis represents a mechanistic phenomenon observed in the context of DES utilization, and its incidence is on the rise. This process is typified by the accretion of lipid-laden foamy macrophages, occasionally accompanied by a necrotic core predominantly localized within the stent deployment region [35].

To offer an at-a-glance perspective, Table 1 schematically shows the cells involved in ISR, as well as the mechanism by which they contribute.

## 3. Risk Factors for ISR

### 3.1. Patient-Related Factors

The clinical prognosticators associated with in-stent restenosis (ISR) encompass a spectrum of factors, comprising, but not limited to, chronic kidney disease, elderly age, male gender, diabetes mellitus, and elevated body mass index, among various others [53]. Furthermore, established cardiovascular risk factors associated with atherosclerosis, such as hypertension, smoking, and dyslipidemia, have been evidenced to exert a pathogenetic influence on the development of neointimal hyperplasia [54].

### 3.2. Clinical Factors

ISR is a multifactorial phenomenon, subject to variability contingent upon individual patient characteristics, angiographic predictors, and the intricacies associated with the angioplasty procedure [55]. Notably, in the context of diabetic patients, it is imperative to underscore that the second generation of DESs exhibit a notable susceptibility to ISR, manifesting with an incidence rate of 8.7% [56]. In contrast, non-diabetic patients present a relatively diminished propensity for ISR, as evidenced by a prevalence of 5.7% [35].

Concerning gender-based prevalence, a study published in 2023 employed intracoronary imaging, specifically, optical coherence tomography (OCT), to demonstrate that the incidence of ISR exhibits a greater frequency among male patients [57]. Hence, the imaging data reveal a notable discrepancy, whereby men exhibit a significantly elevated risk in contrast to women, characterized by a higher incidence of thin-cap fibrous atherosclerosis (TCFA) (37.4% [*n* = 77] vs. 9.3% [*n* = 4], *p* < 0.001), as well as in-stent neo-atherosclerosis (ISNA) (82.0% [*n* = 169] vs. 62.8% [*n* = 27], *p* = 0.005).

Another study, conducted by Bajdechi et al. and published in 2023, explored a distinct patient cohort, specifically, individuals afflicted with Human Immunodeficiency Virus (HIV), revealing their heightened susceptibility to acute coronary events. This elevated risk can be attributed, in part, to the influence of antiviral medications and the concurrent discordant inflammatory response [58]. While patients afflicted with HIV exhibit an elevated risk of recurrent acute coronary syndrome, it is noteworthy that the stage of the disease does not exert a discernible influence on the prevalence of ISR [58].

Arterial hypertension, identified as an independent cardiovascular risk factor [59], was shown for the first time to be responsible for intrastent restenosis in a retrospective study involving 796 patients who underwent angiography due to angina recurrence or reversible myocardial ischemia [60]. The study revealed that effective blood pressure control during the initial PCI procedure correlated with a 24% lower risk of ISR. Additionally, factors such as total cholesterol levels, the use of beta-blockers or antiplatelet agents, and the site of stent implantation were linked to further reductions in ISR, particularly among patients with BMSs [60].

Moreover, it is well established that the cessation of antiplatelet medications ranks among the primary risk factors associated with intrastent restenosis. Consequently, non-adherence to pharmacological treatment regimens represents an additional significant risk factor for the development of ISR [61]. Additionally, individuals presenting with hypertension and a diagnosis of heart failure manifest an elevated susceptibility to ISR [62].

Furthermore, in a retrospective observational study, the incidence of restenosis in the coronary stent group was higher compared to the non-stent group when considering factors such as a family history of CHD, a history of type 2 diabetes, hypertension, smoking, drinking, withdrawal of aspirin, use of conventional doses of statins, calcified lesions, having ≥3 implanted stents, stent length ≥30 mm, and stent diameter <3 mm [63].

### 3.3. Angiographic Factors

**Figure 3:**
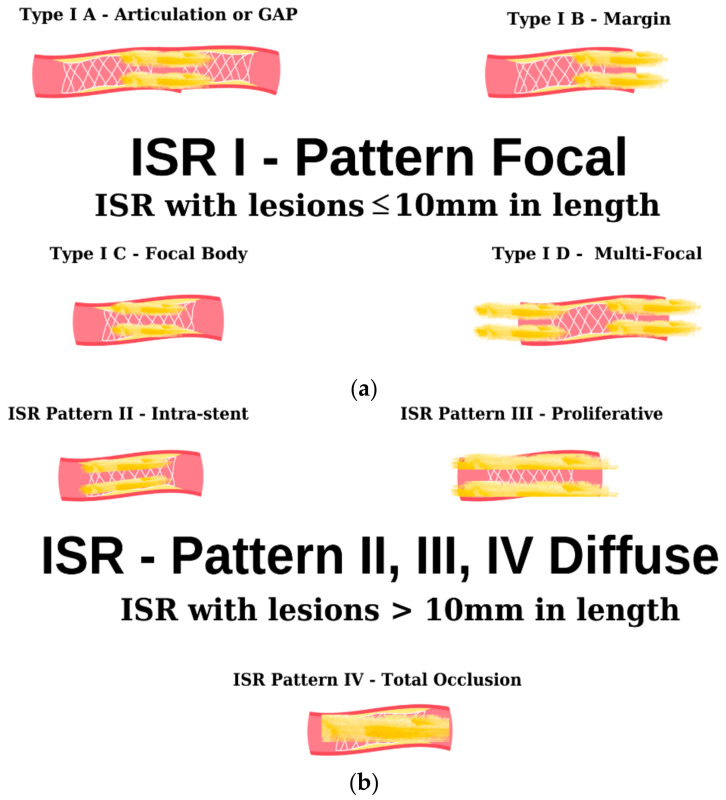
Angiographic classification of intrastent restenosis (adapted from an open-access source [65]). (**a**) Type I: there are four types of focal ISR described in the image above. (**b**) Type II: the observed lesions are confined strictly to the confines of the stent and do not exhibit any extension beyond its proximal or distal extremities. Type III: Diffuse, proliferative ISR. The identified lesions manifest a length exceeding 10 mm, exhibiting an extension that surpasses the boundaries of the stent at both its proximal and distal margins. Type IV: total occlusion of the stent resulting in no coronary perfusion.

As evidenced by the existing literature, the deployment of a stent in a vessel elicits a localized inflammatory response. This inflammatory cascade constitutes a pivotal component in the genesis of a pathological phenomenon recognized as ISR [64]. Furthermore, the intricacies of this multifaceted phenomenon extend beyond the mere presence of a stent, encompassing an array of stent-dependent, intra-stent, and extra-stent risk factors. The schematic delineation portrayed in Figure 3 illustrates the angiographic patterns under discussion. It is imperative to underscore that this classification holds pivotal significance in prognosis and expeditious patient triage for both clinical and investigative objectives [65].

In a retrospective study [66], the utilization of BMSs was associated with an ISR rate of 30%. Subsequently, with the advent of second-generation DESs, this proportion exhibited a notable reduction, declining to 12%. Consequently, the use of BMSs has been identified as a risk factor for ISR, thereby underscoring the rationale behind its diminishing prevalence in contemporary clinical practice in favor of the more favored DESs.

## 4. Clinical Presentation and Diagnosis

In individuals with a prior history of angioplasty involving stent placement, the reappearance of anginal symptoms can stem from several potential factors, including incomplete revascularization (signifying the presence of unresolved lesions), the advancement of underlying atherosclerotic disease, or the occurrence of ISR [67]. Diagnosis is typically confirmed through coronary angiography, and treatment often entails concurrent intervention to address the identified lesions during the same procedure. Although coronary angiography remains the gold standard for diagnosing intrastent restenosis, new data suggest the useful application of coronary CT angiography for diagnosing this pathology. A study published in 2024, which included 102 patients with symptoms raising suspicion of intrastent restenosis, demonstrated that in a large proportion of cases (86.3% of the patients), the CT results, when compared with coronary angiography, were able to exclude ISR (false-negative result) [68]. Considering this finding, we anticipate that in the future, CT scans will additionally assist in the early diagnosis of ISR, facilitating prompt treatment according to current standards.

A notable proportion of patients with bare-metal stents may manifest acute coronary syndrome, with prevalence rates varying from 3% to 20% [69,70]. Furthermore, in two relatively limited-scale investigations, individuals with ISR following the deployment of DESs exhibited clinical symptoms, including unstable angina, in a range between 27% and 50%, and a subset of patients, approximately 5% to 11%, experienced acute myocardial infarction [4,71,72].

### Intravascular Imaging

**Figure 4:**
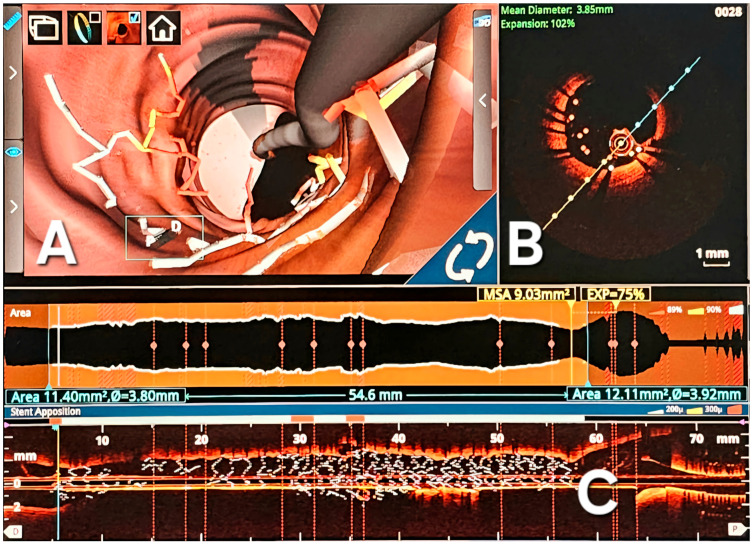
OCT images used for optimal stent optimization. (**A**) Comprehensive three-dimensional intravascular reconstruction, vividly displaying instances of strut malposition; (**B**) Transversal section delineating the specifics of strut malposition, quantifying it at a length of 0.6 mm. This sectional view augments the understanding of the spatial irregularities within the stent deployment. (**C**) Longitudinal 3D reconstruction highlighting the stent along its entire length; significant malposition is observed at the distal end of the stent; D-Longitudinal reconstruction of the stent.

ISR manifests through a complex interplay of diverse mechanical and biological elements, encompassing drug resistance and hypersensitivity reactions to drug polymers [10]. This challenge’s intricate nature necessitates identifying and modifying underlying causative factors. Employing intravascular imaging methods, such as optical coherence tomography (OCT) (Figure 4), or intravascular ultrasound (IVUS), offers a promising avenue for delineating mechanistic intricacies and potentially mitigating the condition [73]. These imaging modalities have notably contributed to elucidating the correlation between specific lesion characteristics and the predisposition to ISR [74]. Nonetheless, the current body of evidence remains insufficient to substantiate the efficacy of intravascular imaging in improving clinical outcomes or forestalling recurrent episodes of ISR [73].

## 5. Clinical Outcomes of ISR

Technological progress, specifically, the advancements in percutaneous coronary interventions, has substantially improved clinical outcomes for acute and chronic coronary syndromes [75]. Presently, the use of new-generation DESs should be considered the gold standard in the treatment of these patient types, regardless of clinical presentation, lesion type, or patient comorbidities [76].

However, the limitations of DESs are related to local inflammatory reactions and late thrombosis [77]. Consequently, alternatives to stent usage have been explored. This has led to the development of Drug-Eluting Balloons (DEBs), which are pharmacologically active and serve both to restore the luminal diameter of the vessel and to deliver drugs without implanting a metal layer in the endothelium [78].

DEBs have emerged as a treatment option for small vessels [79], bifurcations [80], and, recently, ISR [81]. Extensive clinical research has been conducted to demonstrate the utility of DEBs, especially in cases of ISR (Table 2).

When it comes to the chemotherapy used, the coating of balloons with Sirolimus [82] and Paclitaxel [85,86] has been of interest; these have also been compared in clinical studies, with both demonstrating effectiveness [83].

Data suggest the utility of using Paclitaxel-coated balloons in reducing the incidence of restenosis. These findings indicate that local chemotherapy treatment does not necessitate the additional implantation of DESs. The treatment of ISR with Paclitaxel is safe and lowers the rate of needing re-intervention for ISR [87].

Although treating ISR with pharmacologically active balloons has shown satisfactory results in preliminary studies, a recent study highlighted that the treatment of ISR with DEBs had a higher risk of recurrence and major adverse cardiac events (MACEs), compared to patients treated with DESs [88].

Although the treatment of ISR with DEBs appears to have higher rates of MACE compared to DESs, after adjusting for variables, the outcome is similar regarding both strategies [88]. Therefore, using DEBs for ISR can be a supported choice, although further studies are needed to validate the types of lesions for which DEBs are best suited.

## 6. Treatment

Fundamentally, the therapeutic approach to ISR aligns closely with managing stenosis in native coronary arteries. However, it is crucial to emphasize that the presence of an additional stent layer requires increased vigilance and careful consideration during the treatment process [89].

In response to the significance of this issue, a compendium of therapeutic modalities has been contemplated and devised. In comprehensive meta-analyses comparing various treatment modalities, two specific approaches have consistently emerged as superior: the utilization of DESs and drug-coated balloons (DCBs) [90,91]. These two therapeutic options align with the prevailing recommendations outlined in contemporary clinical guidelines [92].

Given the discernible reduction in restenosis rates attributed to the implementation of DESs, these stent types are notably favored in contemporary clinical practice for managing de novo coronary lesions [93]. Furthermore, many meta-analyses consistently position DESs as the primary choice for managing ISR [4,70] contrasting them with alternative interventions such as Paclitaxel-coated balloons [94], intravascular brachytherapy [95,96,97], and conventional balloon angioplasty [98,99,100].

The principal drawback associated with DESs pertains to the enduring presence of a non-absorbable stent scaffold within the vessel. This characteristic can give rise to notable therapeutic complexities, particularly when confronted with a recurrence of ISR. Pharmacologically active balloons are constructed by affixing an active agent onto the surface of a conventional angioplasty balloon [101]. The composition of the balloon coating typically comprises two key constituents: an active lipophilic drug and an excipient, the latter serving to enhance drug solubility and promote its transfer from the balloon surface to the vessel wall [97].

Pharmacologically active balloons offer antiproliferative advantages without necessitating the addition of an extra stent layer, rendering them an appealing alternative for comparison with DESs. Their utility is particularly pertinent in circumstances where the incorporation of an extra stent layer is undesirable, such as in the context of bifurcation lesions or cases involving the presence of multiple stent layers. Moreover, patients who have undergone angioplasty with a pharmacologically active balloon exhibit a differential need for antiplatelet regimens, making them a favored choice, especially in patient populations characterized by an elevated risk of bleeding [81].

Numerous investigations have undertaken comparative assessments between DESs and Paclitaxel-coated drug-coated balloons in treating ISR [102,103,104,105,106,107,108]. Despite the conceptual merits attributed to DCBs, a comprehensive meta-analysis has indicated that the recurrent deployment of DESs for ISR management proves to be more efficacious in reducing target lesion revascularization (TLR) rates at the three-year mark as compared to DCBs [109]. Furthermore, the application of DESs yields superior outcomes concerning the angiographic assessment of residual stenosis. In most comparisons between drug-coated balloons and DESs, these advantages in terms of the angiographic results persist through extended long-term follow-up periods. In individuals experiencing restenosis with bare-metal stents, the available evidence suggests a substantial degree of comparability in both the clinical efficacy and safety profiles between the utilization of DESs and drug-coated balloons [94]. In light of this consideration, the preferred approach in such circumstances involves the utilization of drug-coated balloons, primarily due to the absence of an additional strut layer.

Conversely, in the more complex scenario of ISR in patients with DESs, the deployment of DESs for treatment demonstrates a modestly greater efficacy when compared to the use of DCBs, particularly concerning the requirement for target lesion revascularization [106]. Nevertheless, this increased efficacy necessitates a thorough evaluation of the potential implications of implanting an additional stent layer [89].

Hence, the deployment of either pharmacologically active stents or pharmacologically active balloons warrants consideration in the context of ISR. The selection between these two techniques necessitates careful evaluation based on patient-specific characteristics, the nature of the lesion, underlying risk factors, and the particular stent type involved in the stenosis.

Importantly, to reduce the incidence of ISR, it is advisable to implement a series of strategies. These include appropriate lesion preparation and the utilization of intracoronary imaging to ensure accurate stent apposition to the vessel walls, particularly in the case of complex lesions [110].

In addition to the interventional treatment of ISR, managing risk factors plays a crucial role [63]. Consequently, patients with a history of ISR or previous PCI for coronary artery disease require careful monitoring by their current healthcare provider. Pharmacological treatment should be meticulously guided according to the current medical guidelines [111]. Furthermore, lifestyle modifications and addressing modifiable risk factors are essential for the long-term outcome of these patients [111].

## 7. Conclusions

Despite concerted endeavors and notable advancements, ISR persists as a formidable challenge within the medical domain. The elucidation of novel therapeutic modalities mandates a comprehensive understanding of the intricacies inherent to restenosis, particularly the underlying pathophysiological mechanisms. The imperative lies in consolidating and coordinating the concerted efforts and specialized knowledge dispersed across the global medical community. Such systematic organization is requisite to facilitate the discernment and subsequent formulation of innovative solutions to ameliorate the persistence of restenosis.

Given the significant clinical implications marked by the escalated rates of morbidity and mortality among patients with a prior history of coronary angioplasty featuring stent implantation, research endeavors have primarily pivoted towards the prophylaxis of intrastent restenosis (ISR). Consequently, the advent of successive generations of stents heralded a notable reduction in ISR incidences, albeit its persistent manifestation. Hence, a pragmatic stance necessitates addressing this enduring challenge, propelling prospects toward adopting bioresorbable stents as a promising avenue for mitigation and potentially circumventing ISR’s deleterious consequences.

## Figures and Tables

**Table 1:** Cell types and their role in ISR.

Cell Type	Role in ISR	Mechanism/Effect
Smooth muscle cells	Primary contributors to neointimal hyperplasia	Proliferate and migrate, contributing to luminal narrowing; transition from contractile to synthetic phenotype.
Platelets	Initial responders to stent placement	Release thromboxane A2 and PDGF, inducing oxidative stress and smooth muscle cell transition.
Mast cells	Role in neointimal formation	Release chymase, influencing angiotensin II and TGF-β production, leading to fibroblast proliferation.
Monocytes	Involved in the inflammatory response and late ISR risk	Elevated levels post-PCI are indicative of late ISR risk. Participate in cytokine secretion.
Eosinophils	Associated with late ISR	Elevated levels post-PCI are predictive of late ISR.
Macrophages	Part of the inflammatory response	Infiltrate subendothelial space, involved in neo-atherosclerosis and neointimal formation.
Endothelial cells	Affected by stent deployment	Disruption leads to exposure of the intimal layer and a prothrombotic effect.
Bone marrow progenitor cells (BMPCs)	Contribute to neointimal formation	Recruitment and proliferation within the extracellular matrix.
Fibroblasts	Involved in neointimal formation	Proliferation influenced by mast cell-released chymase and TGF-β.

**Table 2:** Clinical trials focused on DEBs.

ClinicalTrials.gov Identifier	Official Title	Intervention/Treatment	References
NCT04280029	SELUTION SLR™ 014 In-stent Restenosis	Device: SELUTION SLR™ DEB; Device: Control	[82]
NCT03667313	Treatment of In-Stent Restenosis 2 Study	Combination Product: sirolimus-eluting balloon (SEB) Magic Touch Combination Product: paclitaxel-eluting balloon (PEB) Sequent Please	[83]
NCT03242096	Treatment of Coronary In-stent Restenosis (ISR) by a Sirolimus Coated or a Paclitaxel Coated Balloon	Combination Product: Sirolimus-coated balloon Combination Product: Paclitaxel-coated balloon	[84]
NCT00106587	Treatment of In-Stent Restenosis by Paclitaxel Coated PTCA Balloons (PACCOCATH—ISR I)	Device: PTCA Combination Product: Paclitaxel-coated balloon catheter (device with drug)	[85]
NCT00409981	Treatment of in-Stent Restenosis by Paclitaxel Coated PTCA Balloons (PACCOCATH—ISR II)	Device: Paclitaxel-coated balloon catheter (device with drug)	[86]

## Data Availability

Not applicable.
